# Importance of the Precautionary Principle With Regard to the Risk of Exposure to Aerosols Containing Viral Loads of SARS-CoV-2 Present in Feces: In Perspective

**DOI:** 10.3389/fpubh.2022.892290

**Published:** 2022-05-25

**Authors:** Richard Massicotte, Mafu Akier Assanta, Kakese Mukosa Rosette

**Affiliations:** ^1^Laboratory of Innovation and Analysis of Bioperformance, Ecole Polytechnique de Montreal, Montreal, QC, Canada; ^2^Food Research and Development Centre, Agriculture and Agri-Food Canada, Saint-Hyacinthe, QC, Canada; ^3^Groupe de Recherche sur les Maladies Infectieuses du Porc, Département de Pathologie et Microbiologie, Faculté de Médecine Vétérinaire, Centre de Recherche d'Infectiologie Porcine et Avicole, Université de Montréal, Saint-Hyacinthe, QC, Canada

**Keywords:** COVID-19, aerosol, toilet, emission, exposure

## Abstract

In COVID-19 infection, the emissions of droplets and aerosols produced by the respiratory tract of contaminated subjects may represent a high risk of spreading the SARS-COV-2 virus in the environment. Thus, studies have shown that there is, at least, another source of droplets and aerosols in which viral particles of SARS-COV-2 can be found. It happens after flushing of toilet to dispose of the stools of a patient who has contracted COVID-19. The presence of viral particles of SARS-COV-2 in the stool could be linked to the concentration of angiotensin-converting enzyme 2 (ACE2) found on the surface of intestinal cells. Therefore, there is a reason to wonder whether the emission of viral particles by activating a toilet flush could represent an important potential risk of contamination for health care workers. To investigate this hypothesis, we have correlated different studies on the production of droplets and aerosols as well as the presence of viral particles following flush of toilet. This pooling of these studies led to the following conclusion: the precautionary principle should be applied with regard to the potential risk represented by viral particles of SARV-COV-2 in the stool when flushing the toilet.

## Introduction

The SARS-CoV-2 virus, which caused the COVID-19 pandemic, is an enveloped single-stranded RNA virus that is a type of coronavirus. It is 80% genetically similar to SARS-CoV, which caused the severe acute respiratory syndrome (SARS) epidemic in 2002 (1, 2). The main recognized mode of transmission of this type of virus is the diffusion of particles in the form of contaminated droplets from the respiratory system. During the 2002 SARS epidemic, researchers found viral particles of the virus in the stool of infected people such as 2002 SARS,SARS-CoV-2 viral particles found in the stool of infected people (3–8). The presence of SARS-COV-2 viral particles in the stool has raised the hypothesis of a possible fecal–oral contamination route (5, 9). In addition, the Institut National d'Excellence en Santé et Services Sociaux in Quebec, Canada (10), conducted a review of the literature on COVID-19 and gastrointestinal symptoms. This organization has come to the conclusion that a small proportion of people (on average 7.8%) with COVID-19 may have gastrointestinal symptoms. For their part, Pan et al. (11) found that about 17% of cases with gastrointestinal problems presented with diarrhea or had loose stool. Now, the Centers for Disease Control and Prevention (CDC) also considers diarrhea a symptom of COVID-19 (12). The cause of the diarrhea may be related to the invasion of enterocytes that have angiotensin-converting enzyme 2 (ACE2) on their surface. These proteins promote binding with SARS-CoV-2 (13, 14). The presence of ACE2 would particularly favor the infection of enterocytes by coronaviruses (15). This infection would alter the functions of the intestinal mucosa, thus causing diarrhea.

**Figure 1:**
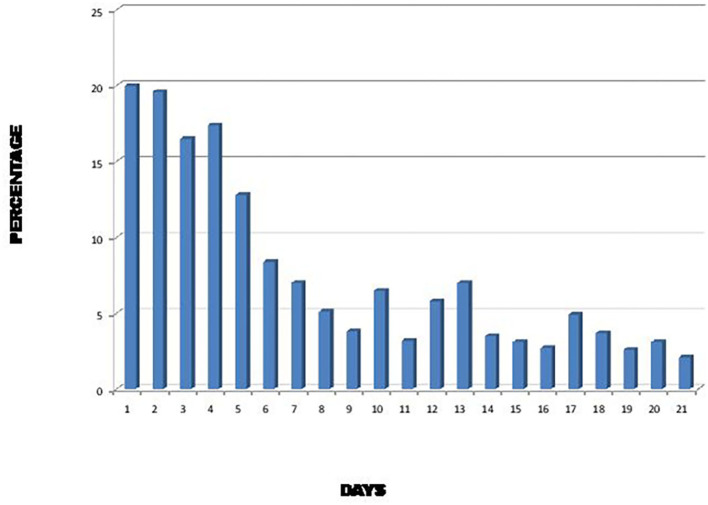
Percentage of SARS patients with diarrhea during the first 21 days of illness (16).

During the SARS epidemic in 2002, it was found that diarrhea was more frequent during the first 6 days of illness and could last for 21 days after the onset of symptoms (Figure 1) (16). As SARS-CoV-2 is genetically closely related to the 2002 SARS virus (17, 18), it is therefore not surprising that SARS-CoV-2 viral particles can be found in the stool of patients infected with this virus several days after the disappearance of respiratory symptoms (8).

## The Issue

The presence of SARS-CoV-2 viral particles in feces raises important questions about the risk that handling stool poses to healthcare workers. First, there is a potential danger of splashing with liquid stool when transporting bedpans to empty them. Second, there is also a potential risk of exposure when the stool is in the toilet. This threat is believed to come from the creation of droplets and aerosols laden with SARS-CoV-2 virus when the toilet is flushed. There is therefore a risk of possible exposure by aerosol for the general population in public toilets (19, 20) as well as the healthcare workers caring for patients with COVID-19 Despite the context of the pandemic, this risk of contamination for health personnel is not generally considered by public health authorities because it is not a proven risk, which would require the implementation of preventive procedures. Although this contamination risk has not yet been proven, the various studies carried out so far tend to show that this path presents a plausible danger of contamination.

Johnson et al. (21) characterized, by size, the emissions spread by three different types of toilets when flushed. They observed that the emissions in the form of droplets, meaning having a diameter of 5 μm and more, constitute the smallest quantity of particles emitted, regardless of the model of toilet, compared to aerosols (smaller than 5 μm). Because of their size, the droplets fall quickly on neighboring surfaces, including sinks, faucets and toilet paper dispensers. These droplets thus present a risk of contamination by indirect contact (22, 23). Depending on the environmental conditions, if the surfaces are not adequately cleaned and disinfected, there is the possibility of to have an airborne resuspension of the virus.

According to the results of Johnson et al. (21), all of the emissions produced when a toilet is flushed end up in the form of an aerosol. This observation is corroborated by Knowlton (24). In light of these observations, potentially contaminated aerosols that came from a toilet may remain suspended in the air for a longer time and therefore may travel longer distances, depending on environmental conditions. It is important to emphasize that the environmental conditions can not only favor the agglomeration of the particles but also allow them to contract and dry out (25), thus making it possible for them to remain suspended in the air for longer. Eventually, they will settle on a surface and, if the virus is still viable, the surface may become a potential vehicle for indirect transmission some distance from the emission source (23, 26, 27).

**Figure 2:**
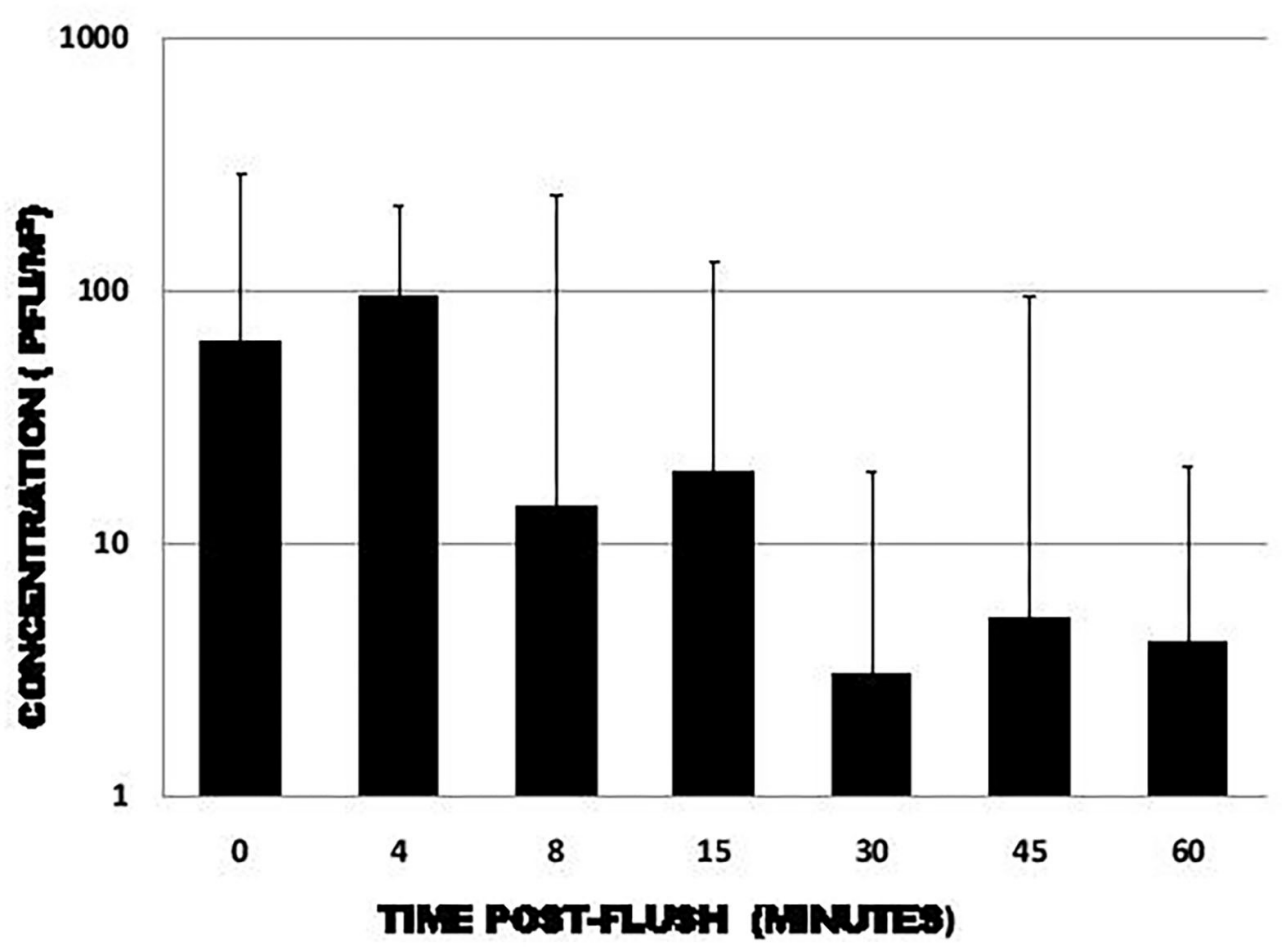
Average concentration of MS2 phases per m^3^ as a function of time after one flush (28).

Using the MS2 phage, Cooper (28) studied the presence of viral particles in an aerosol generated by a flushing toilet in a bathroom. The results of the study showed that the concentration of the phage in the air was high 4 min after the toilet was flushed (Figure 2). After 1 h, the viral load was reduced ~20-fold. It is important to note that the results of Cooper (28) were obtained using a single toilet flush in an environment where there were about 10 air exchanges per hour.

**Figure 3:**
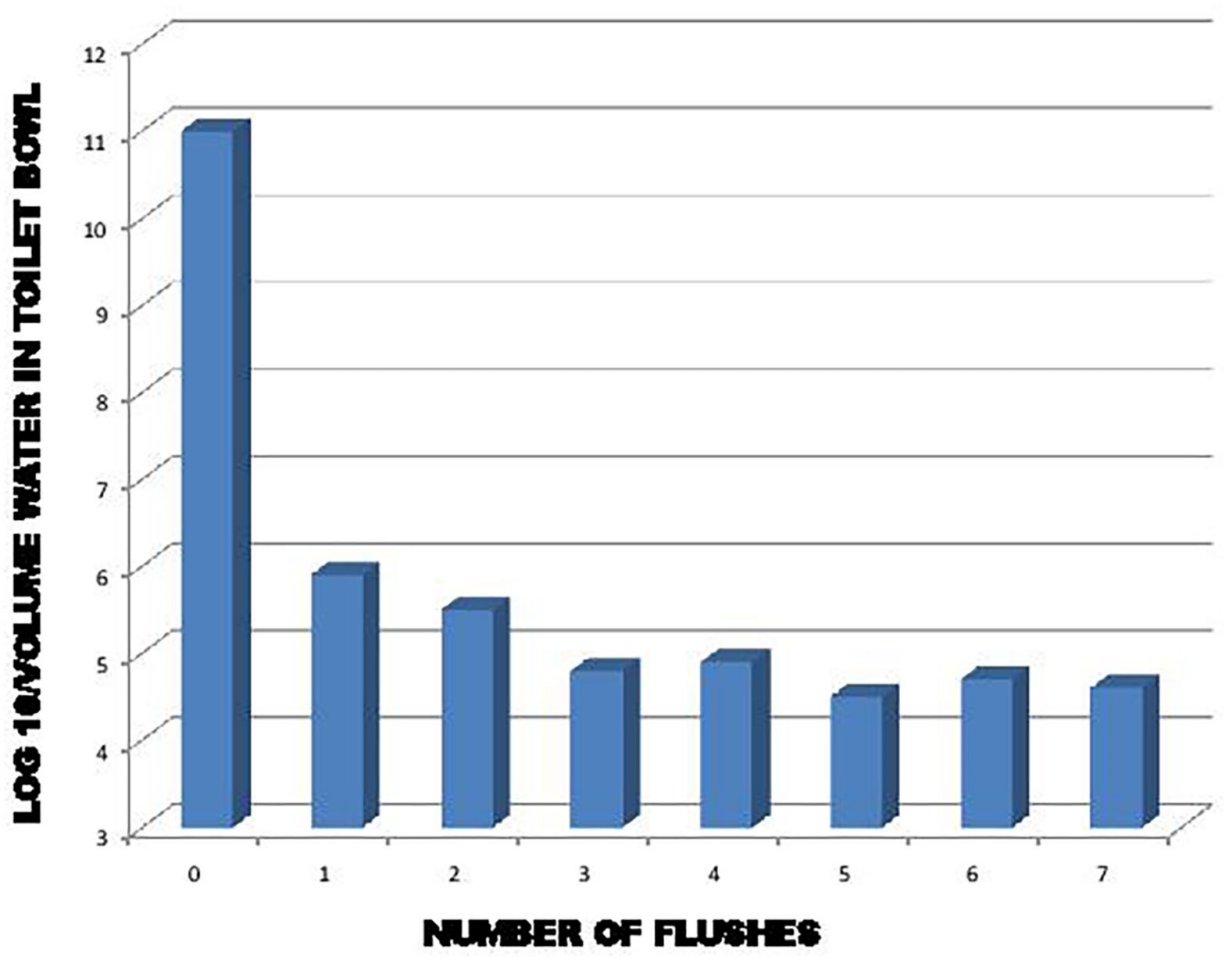
Effect of flushing on removal of exogenously added bacteria and viruses to the toilet bowl (22).

It is interesting to make a connection between the studies of Gerba et al. (22), Cooper (28), and Barker and Jones (29). Gerba et al. (22) and Barker and Jones (29) demonstrated that a significant amount of virus remained on toilet surfaces after a single flush. Some of this viral load that has remained in the toilet can be suspended by a few toilet flushes afterwards, as shown in Figure 3. As demonstrated by Cooper (28), an emission of viral particles is found suspended in the air for some time in the form of droplets and aerosols after the toilet has been flushed. Hypothetically, if there is a virus in the toilet, the frequency of toilet flushing could play a role in maintaining a certain SARS-CoV-2 viral load in the air for a longer period of time. By correlation, this would thus promote an increase or to keep of the concentration of the virus in the air, in addition to increasing the risk of contamination of the various surfaces present, all depending on the environmental conditions.

## Discussion

During this pandemic period, the issue of aerosols is particularly worrying, because viable viral particles of SARS-CoV-2 have been found in stool (8) and toilets are sources of droplet and aerosol emissions laden with viral fecal particles (24, 28). These particles emitted when the toilet is flushed could contribute to the environmental viral load of SARS-CoV-2. Some of this load is found in the air for a while in particles, for which the size modulates the distribution of the virus throughout the respiratory tract (30) when they are inhaled. This means that if viable particles of the virus contained in aerosols end up in the lungs, they would be a potential source for the onset of COVID-19 symptoms. There is an abundance of ACE2 proteins in the lungs (2020). It should be noted that this virus may have a low infectious dose (31) and it can survive for hours in aerosols and on surfaces it is deposited on, depending on environmental conditions and the type of surface (32).

Still, the presence of viable particles of this type of virus appears to be in frequent in the stool, and the survival rate is unknown due to environmental stress when the particles are released into the environment (33). The virulence of the pathogen, the environmental load present, the ability of the pathogen to spread, and the vulnerability of the host mean that SARS-CoV-2, in the form of droplets and aerosols from toilets, presents a potential infectious risk that should be considered in health care settings. Due to the level of uncertainty surrounding this risk, it is currently not considered to be a proven, known risk. In this context, it is difficult to apply prevention principles that seek to reduce the risks associated with an established and recognized danger.

The failure to apply the precautionary principle today is of particular concern because in 1985 (34) demonstrated for the first time that a coronavirus present in an aerosol can survive at least 3 days in the air according to the environmental conditions.

This concern applies, among other things, when flushing a toilet containing contaminated stool, emissions of droplets and aerosol contaminated with SARS-COV-2 are produced. As we found with the work of Gerba et al. (22), the toilet must be flushed at least three times to adequately reduce the risk of contaminated emissions. Knowing that each time we operate the toilet there is formation of aerosol which remains in suspension for a certain time. Therefore, environmental microbial and viral load increase according to the frequency of action of the toilet and of the traffic of these.

These emissions could therefore represent a potential infectious risk for people in the environment of the emitting source. Admittedly, the entire population is exposed to this risk, but for healthcare personnel, the presence of potentially contaminated emissions from toilets can add to the environmental load from the respiratory tract of patients with COVID-19 (35, 36). According to Birgand et al. (37), the degree of air contamination by SARSCoV-2 in hospitals reported to be positive for 24% of air samples from toilets with an average of viral RNA concentrations per m^3^ air higher than that of any other area sampled. This increase in the environmental load also represents an increase in the risk of contamination for personnel.

However, if it is desired to reduce a hypothetical but plausible potential risk, a proactive approach can be adopted by applying the precautionary principle. Because of the evolution of the COVID-19 pandemic and the lack of knowledge, it would be logical and rational to implement preventive precautions to reduce the risks of exposure of healthcare personnel to SARS-CoV-2.

## Conclusion

The originality of this article comes from the fact that it combines the results of different works relating to the characterization of the emissions produced when we flush the toilet, the number of times we have to flush the toilet to significantly reduce the risk of infection as well as the variation in the concentration of the virus in the air as a function of time. Combination of these information linked to the results of work carried out more recently by various researchers (19, 20, 37, 38) which demonstrated the presence of SARS-COV-2 in emissions from toilets clearly indicates that the risk of exposure to aerosols contaminated by SARS-COV-2 can persist for some time, even in the absence of contaminated stools in the toilet.

Hypothetically, with regard to ventilation, the dispersion of SARS-COV-2 in aerosol form could exceed the limits of the bathroom. Therefore, particular attention should be paid to this source of potential contamination and especially in a hospital environment by applying the precautionary principle, i.e., applying stricter protective measures.

However, studies have not yet demonstrated a direct cause and effect link between the aerosols produced when we activate a toilet flush and cases of contamination of people (39). This lack of proven evidence does not encourage public health authorities to recommend stricter protective measures for healthcare workers. Hence extensive research in line with this finding should be carried out in the future. However, in view of current knowledge to limit the risks of contamination in the absence of infection prevention protocols precautionary principle is an important avenue to proactively consider. Compliance with this principle will help reduce exposure to the potential infectious risk of SARS-CoV-2 in aerosol form in the healthcare environment.

## Data Availability Statement

The figures presented in this study are based on those find in the corresponding references. The further inquiries can be directed to the corresponding author.

## Author Contributions

RM contributed to the conception of the work. MA contributed to revised the paper. KR contributed to the supervision of the manuscript. All authors contributed to the article and approved the submitted version.

## Conflict of Interest

The authors declare that the research was conducted in the absence of any commercial or financial relationships that could be construed as a potential conflict of interest.

## Publisher's Note

All claims expressed in this article are solely those of the authors and do not necessarily represent those of their affiliated organizations, or those of the publisher, the editors and the reviewers. Any product that may be evaluated in this article, or claim that may be made by its manufacturer, is not guaranteed or endorsed by the publisher.
